# Overtraining is associated with DNA damage in blood and skeletal muscle cells of Swiss mice

**DOI:** 10.1186/1472-6793-13-11

**Published:** 2013-10-08

**Authors:** Bruno Cesar Pereira, José Rodrigo Pauli, Lusânia Maria Greggi Antunes, Ellen Cristini de Freitas, Mara Ribeiro de Almeida, Vinícius de Paula Venâncio, Eduardo Rochete Ropelle, Claudio Teodoro de Souza, Dennys Esper Cintra, Marcelo Papoti, Adelino Sanchez Ramos da Silva

**Affiliations:** 1 Universidade de São Paulo (USP), Escola de Educação Física e Esporte de Ribeirão Preto (EEFERP), Ribeirão Preto, São Paulo, Brasil; 2 Universidade Estadual Paulista (UNESP), Curso de Pós-graduação em Ciências da Motricidade Humana, Rio Claro, São Paulo, Brasil; 3 Universidade de São Paulo (USP), Faculdade de Ciências Farmacêuticas de Ribeirão Preto, Departamento de Análises Clínicas Toxicológicas e Bromatológicas, Ribeirão Preto, São Paulo, Brasil; 4 Universidade Estadual de Campinas (UNICAMP), Faculdade de Ciências Aplicadas, Curso de Pós-graduação em Nutrição, Esporte e Metabolismo, Limeira, São Paulo, Brasil; 5 Universidade do Extremo Sul Catarinense, Laboratório de Bioquímica e Fisiologia, Criciúma, Santa Catarina, Brasil

**Keywords:** DNA damage, Aerobic training, Overtraining, Oxidative stress

## Abstract

**Background:**

The alkaline version of the single-cell gel (comet) assay is a useful method for quantifying DNA damage. Although some studies on chronic and acute effects of exercise on DNA damage measured by the comet assay have been performed, it is unknown if an aerobic training protocol with intensity, volume, and load clearly defined will improve performance without leading to peripheral blood cell DNA damage. In addition, the effects of overtraining on DNA damage are unknown. Therefore, this study aimed to examine the effects of aerobic training and overtraining on DNA damage in peripheral blood and skeletal muscle cells in Swiss mice. To examine possible changes in these parameters with oxidative stress, we measured reduced glutathione (GSH) levels in total blood, and GSH levels and lipid peroxidation in muscle samples.

**Results:**

Performance evaluations (i.e., incremental load and exhaustive tests) showed significant intra and inter-group differences. The overtrained (OTR) group showed a significant increase in the percentage of DNA in the tail compared with the control (C) and trained (TR) groups. GSH levels were significantly lower in the OTR group than in the C and TR groups. The OTR group had significantly higher lipid peroxidation levels compared with the C and TR groups.

**Conclusions:**

Aerobic and anaerobic performance parameters can be improved in training at maximal lactate steady state during 8 weeks without leading to DNA damage in peripheral blood and skeletal muscle cells or to oxidative stress in skeletal muscle cells. However, overtraining induced by downhill running training sessions is associated with DNA damage in peripheral blood and skeletal muscle cells, and with oxidative stress in skeletal muscle cells and total blood.

## Background

The alkaline version of the single-cell gel (comet) assay is considered as a method for quantifying DNA damage [1,2]. Animal experiments have shown chronic and acute effects of exercise on DNA damage as measured by the comet assay [1,3-5]. Selman et al. [5] showed that short-term voluntary wheel running (i.e., 1 and 7 days) does not lead to lymphocyte DNA damage. In addition, recently, Siu et al. [3] observed that after 8 and 20 weeks of habitual voluntary exercise (i.e., wheel running) the percentage of DNA content in the tail diminished by 21% and 45%, respectively, compared with sedentary control rats. The authors suggested that elevated expression of antioxidant enzymes and DNA-repairing enzymes play a fundamental role in the protective effect of habitual exercise on oxidant-induced lymphocyte DNA damage.

For studying exercise physiology and sports medicine, besides the lack of lymphocyte DNA damage, defining the chronic exercise load (i.e., intensity *versus* volume) and determining if this specific exercise leads to positive performance adaptations, such as development of aerobic capacity, are important. Studies on habitual voluntary exercise with wheel running [3,5] provide no information regarding training load or performance development. Recently, Martins et al. [1] used a swimming training protocol proposed by Gobatto et al. [6], and observed that male adult Wistar rats had diminished DNA damage expressed as the mean percentage of tail DNA in heart cells treated with doxorubicin, an anti-tumor drug that leads to cardiotoxicity [7].

These authors also verified that trained rodents treated with saline do not have DNA damage in cells of the heart, kidney, and liver compared with sedentary rodents [7]. However, Martins et al.’s investigation [1] did not show results on changes in performance. Martins et al.’s study [1] was the first investigation to determine the responses of DNA damage as measured by the comet assay to a training protocol with intensity, volume and load clearly defined. However, previous studies [1,3-5] on the effects of exercise on DNA damage did not examine whether an aerobic training protocol with intensity, volume and load clearly defined improves performance without leading to peripheral blood cell DNA damage. Furthermore, although these previous studies investigated exercise-induced DNA damage [1,3-5], none of them provided information on DNA damage in skeletal muscle cells.

In a treadmill running study, Wierzba et al. [4] observed that a 50-min single bout with intensity corresponding to 75–85% of maximal oxygen uptake (VO_2_max) leads to lymphocyte DNA damage compared with sedentary rodents. Notably, 33–35 min of the acute exercise bout were performed at an intensity above the lactate threshold. Therefore, the authors considered that a strenuous single bout of exercise is relevant for induction of lymphocyte DNA damage [4]. In addition, using the protocol described by Hohl et al. [8], Dong et al. [9] showed that overtrained rats had lymphocyte DNA damage.

Meeusen et al. [10] defined functional overreaching (FOR) as a short-term performance decrement without severe psychological or other lasting negative symptoms that eventually leads to improvement in performance after days of recovery. Furthermore, the authors characterized nonfunctional overreaching (NFOR) as a performance decrement that can be reversed after weeks or months of recovery, while a performance decrement in overtraining syndrome (OTS) can last months to years. Recently, we have developed a new overtraining protocol based on downhill running sessions that lead to NFOR in 100% of mice [11].

The present study aimed to examine the effects of aerobic training and overtraining protocols on DNA damage to peripheral blood and skeletal muscle cells in Swiss mice. To examine possible changes in peripheral blood and skeletal muscle cell DNA with oxidative stress, we measured reduced glutathione (GSH) levels in total blood, and GSH levels and lipid peroxidation in gastrocnemius samples in trained and overtrained mice.

## Results

### Body weight and food intake

**Figure 1:**
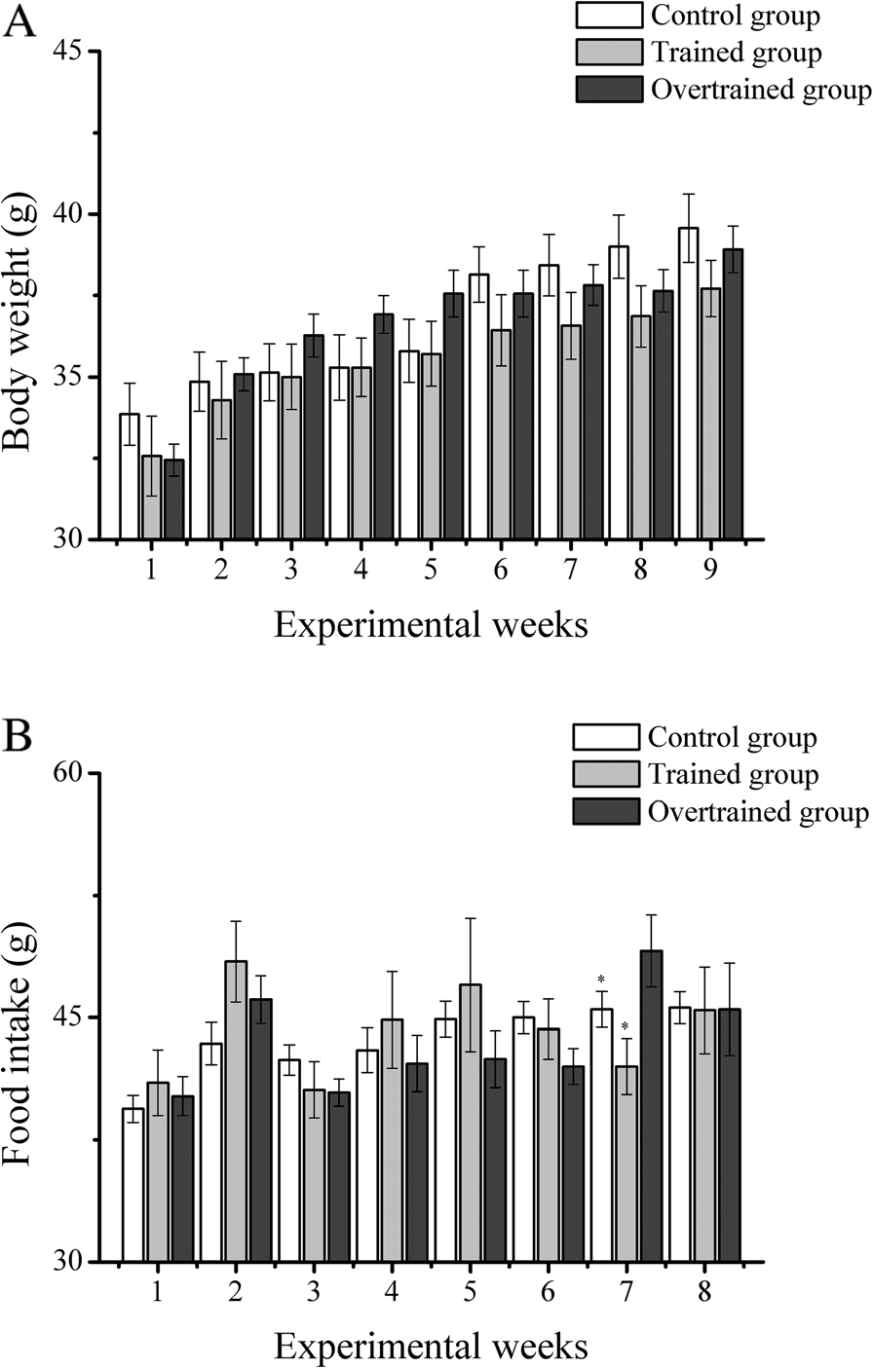
Body weight (A) and food intake (B) in the control, trained, and overtrained groups. ^*^Statistical difference from the overtrained group for the same experimental week.

Body weight in the control, trained, and overtrained groups was not significantly different during the experimental weeks (Figure 1A). The control and trained groups showed a significant decrease in food intake compared with the overtrained group at the end of week 7 (45.5±1.1 and 42.0±1.7 g *versus* 49.1±2.2 g, Figure 1B).

### Incremental load test

**Figure 2:**
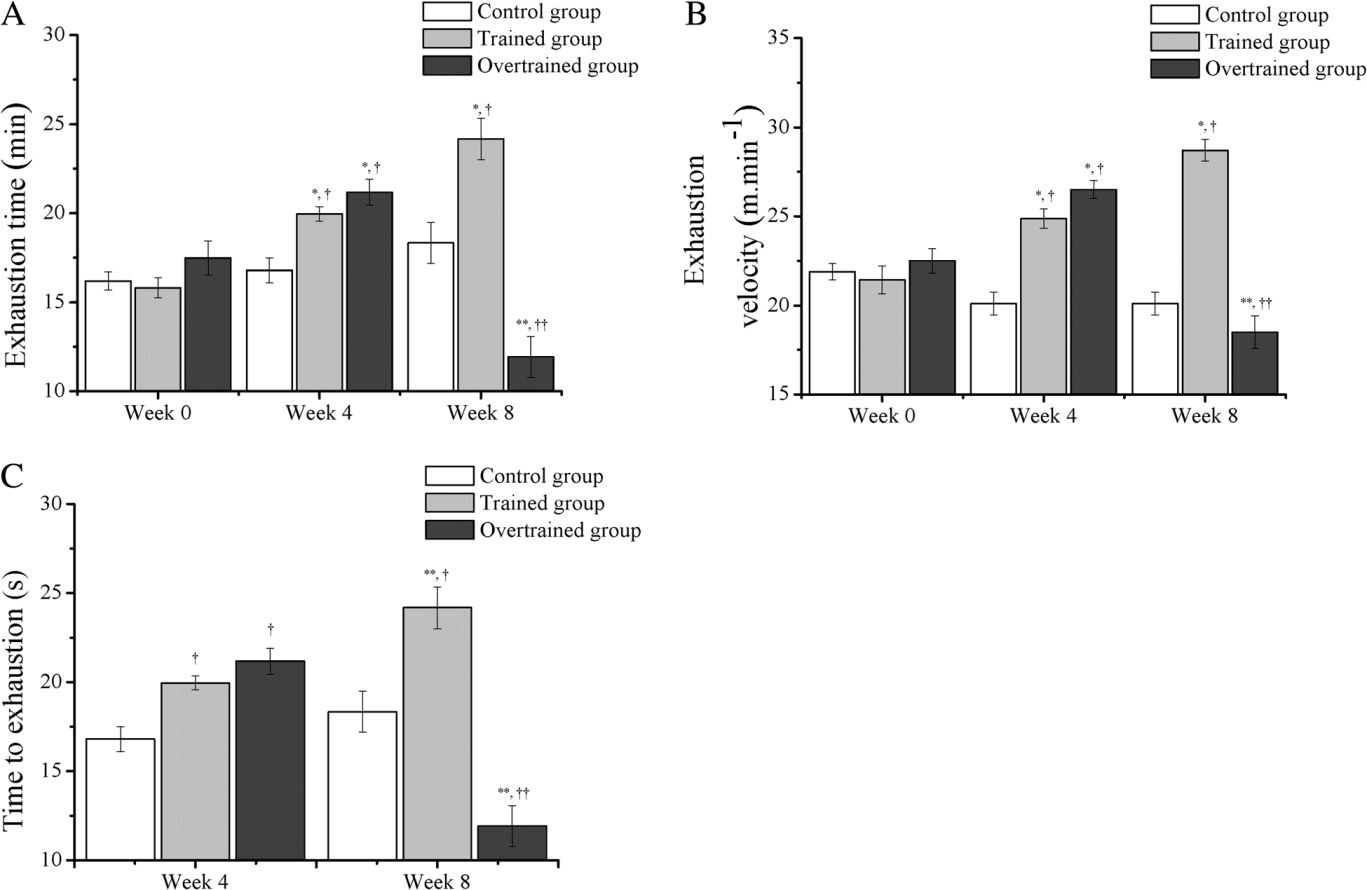
Exhaustion time (A) and velocity (B), and time to exhaustion (C) during the experimental period in the control, trained and overtrained groups. ^*^Statistical difference from week 0 within the same experimental group. ^**^Statistical difference from week 4 within the same experimental group. ^†^Statistical difference from the control group for the same training period. ^††^Statistical difference from the trained group for the same training period.

With regard to inter-group differences (Figure 2A and 2B), the exhaustion time (s) and velocity (m.min^-1^) in the trained group were significantly higher compared with that in the control group at week 4 (20.0±0.4 min and 24.9±0.6 m.min^-1^*versus* 16.2±0.5 min and 21.9±0.5 m.min^-1^) and week 8 (24.2±1.2 min and 28.7±0.6 m.min^-1^*versus* 18.3±1.2 min and 20.1±0.6 m.min^-1^). The exhaustion time and velocity in the overtrained group were significantly higher than those in the control group at week 4 (21.2±0.7 min and 26.5±0.5 m.min^-1^*versus* 16.2±0.5 min and 21.9±0.5 m.min^-1^). Furthermore, the exhaustion time and velocity in the overtrained group were significantly lower than those in the trained group at week 8 (11.9±1.2 min and 18.5±0.9 m.min^-1^*versus* 24.2±1.2 min and 28.7±0.6 m.min^-1^).

With regard to intra-group differences (Figure 2A and 2B), exhaustion time and velocity in the trained group were increased at week 4 (20.0±0.4 min and 24.9±0.6 m.min^-1^) and week 8 (24.2±1.2 min and 28.7±0.6 m.min^-1^) compared with week 0 (15.8±0.6 min and 21.4±0.8 m.min^-1^). However, while exhaustion time and velocity in the overtrained group were increased at week 4 (i.e. 21.2±0.7 min and 26.5±0.5 m.min^-1^) compared with week 0 (17.5±1.0 min and 22.5±0.7 m.min^-1^), these parameters were decreased at week 8 (11.9±1.2 min and 18.5±0.9 m.min^-1^) compared with week 4 (21.2±0.7 min and 26.5±0.5 m.min^-1^).

### Exhaustive test

With regard to inter-group differences (Figure 2C), the time to exhaustion in the trained group was significantly higher than that in the control group at week 4 (39.3±2.6 s *versus* 23.4±1.3 s) and week 8 (71.7±5.7 s *versus* 23.2±1.3 s). The time to exhaustion in the overtrained group was significantly higher than that in the control group at week 4 (43.8±3.0 s *versus* 23.4±1.3 s). In addition, the time to exhaustion in the overtrained group was significantly lower than that in the trained group at week 8 (19.7±2.6 s *versus* 71.7±5.7 s). With regard to intra-group differences (Figure 2C), the time to exhaustion was increased in the trained group at week 8 compared with week 4 (71.7±5.7 s *versus* 39.3±2.6 s). However, the time to exhaustion was decreased in the overtrained group at week 8 compared with week 4 (19.7±2.6 s *versus* 43.8±3.0 s).

### Percentage of DNA in the tail from peripheral blood and skeletal muscle cells

**Figure 3:**
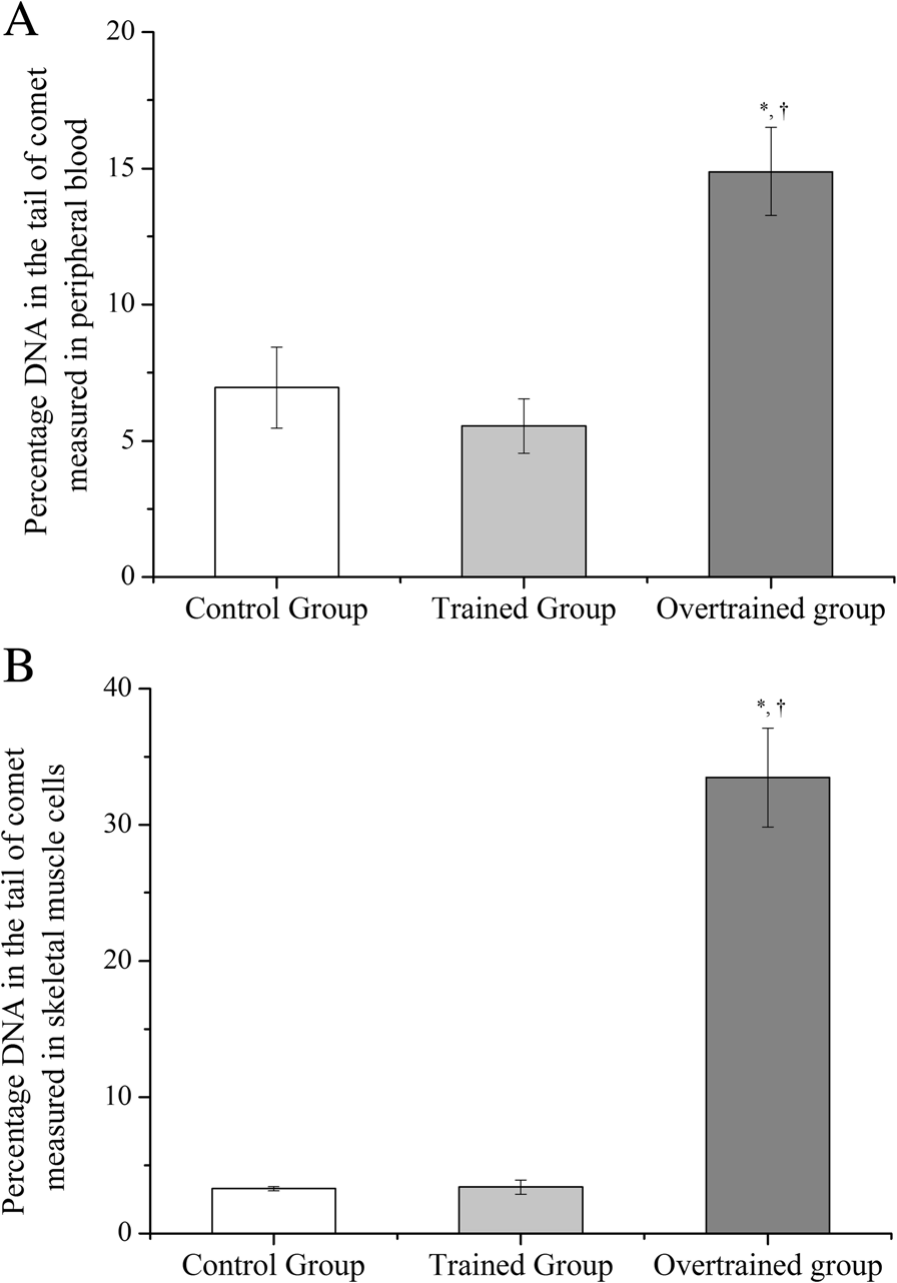
Percentage DNA from peripheral blood (A) and skeletal muscle cells (B) in the experimental groups. ^*^Statistical difference from the control group. ^†^Statistical difference from the trained group.

The overtrained group had a significantly higher percentage of DNA in the tail compared with the control and trained groups in peripheral blood (14.9±1.6 *versus* 6.95±1.5 and 5.55±1.0, B) and skeletal muscle cells (i.e. 33.5±3.6 *versus* 3.3±0.2 and 3.4±0.5, Figure 3A and 3B). Independent from the experimental group, the comet assay scores measured in blood and skeletal muscle cells showed a significant correlation (r=0.75).

### GSH levels in skeletal muscle cells and total blood, and thiobarbituric acid reactive substance (TBARS) levels in skeletal muscle cells

**Figure 4:**
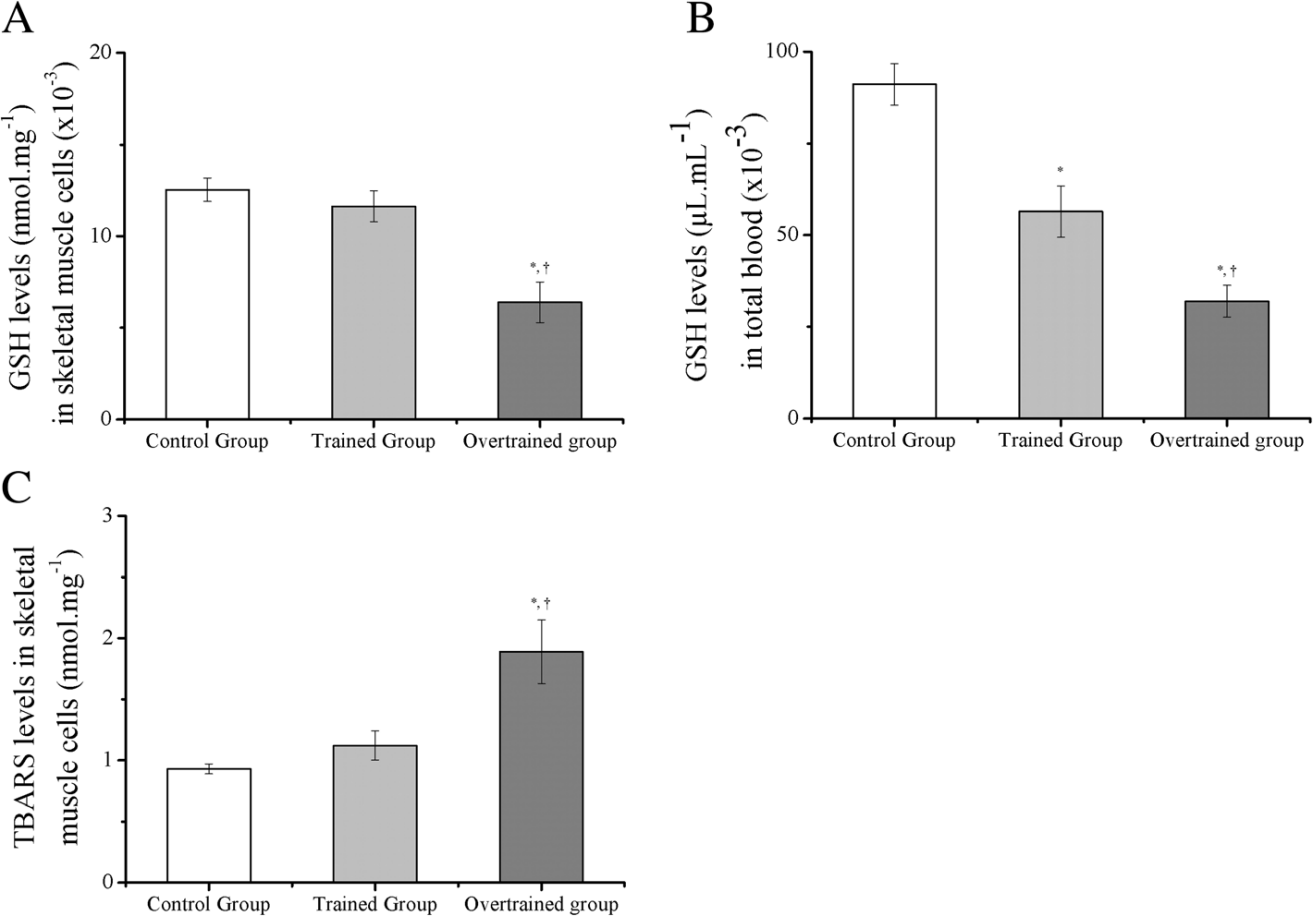
GSH levels in skeletal muscle cells (A) and total blood (B), and TBARS levels in skeletal muscle cells (C) in the control, trained and overtrained groups. ^*^Statistical difference from the control group. ^†^Statistical difference from the trained group.

GSH levels in skeletal muscle cells were significantly lower in the overtrained group than in the control and trained groups (6.4±1.1 nmol.mg^-1^ × 10^-3^*versus* 12.5±0.6 and 11.6±0.9 nmol.mg^-1^ × 10^-3^, Figure 4A). GSH levels in total blood in the trained group were significantly lower than those in the control group (56.4±7.0 μmol.mL^-1^ × 10^-3^*versus* 91.1±5.7 μmol.mL^-1^ × 10^-3^). In addition, the overtrained group had significantly lower GSH levels in total blood than did the control and trained groups (31.9±4.3 μmol.mL^-1^ × 10^-3^*versus* 91.1±5.7 and 56.4±7.0 μmol.mL^-1^ × 10^-3^, Figure 4B). Independent from the experimental group, GSH levels measured in skeletal muscle cells and total blood were significantly correlated (r=0.77). The overtrained group had significantly higher levels of TBARS in skeletal muscle cells than did the control and trained groups (1.9±0.3 nmol.mL^-1^*versus* 0.9±0.1 and 1.1±0.1 nmol.mL^-1^, Figure 4C).

## Discussion

The main findings of the present investigation were that: 1) the training protocol proposed by Ferreira et al. [12] improved performance (i.e., exhaustion time and velocity, and time to exhaustion) in mice without leading to DNA damage in peripheral blood and skeletal muscle cells or oxidative stress in skeletal muscle cells; and 2) the overtraining protocol based on downhill running sessions [11] led to DNA damage in peripheral blood and skeletal muscle cells, and to oxidative stress in skeletal muscle cells and total blood.

In the present study, we did not observe any significant differences in body weight between the groups during the experimental weeks. However, mice in the overtrained group had significantly higher food intake at the end of week 7 compared with that in the control and trained groups. Food intake at week 7 represents the amount of food that mice eat from weeks 7 to 8. Based on significant correlations from percentage changes in food intake and body gain from weeks 7 to 8 (data not shown), we hypothesize that these mice increased their food intake to compensate for energetic demand after the 3-week period of downhill running sessions.

Although our experimental procedures were exactly the same as those by Pereira et al. [11], our findings of body weight and food intake were not similar. Armstrong and VanHeest [13] considered the loss of body weight as one of the signs and symptoms related to overtraining. Future research using these protocols needs to increase the number of days that body weight and food intake are recorded from one per week to three or five per week, and metabolic cages should be used to better control these specific variables.

In contrast to the discrepancies between the present study and Pereira et al.’s study [11] regarding body weight and food intake, our results on incremental load and exhaustive tests were similar in the trained and overtrained groups to their study. After training during for 8 weeks in intensity corresponding to a maximal lactate steady state (MLSS; i.e., 60% of exhaustion velocity [EV]) [12], mice improved their exhaustion time, exhaustion velocity, and time to exhaustion by approximately 34.0%, 52.7%, and 21.4%, respectively. As previously demonstrated by Pereira et al. [11], the improvement in incremental load test parameters occurred mainly during the first 4 weeks of aerobic training, and these variables were not significantly different from weeks 4 to 8.

MLSS represents the gold standard protocol for identification of the metabolic aerobic/anaerobic transition point during exercise [14,15]. In our study, the intensity of the exhaustive test (i.e., 36 m.min^-1^) was 141.6% and 109.3% higher than that corresponding to MLSS in week 4 (i.e., 60% of EV=14.9 m.min^-1^) and week 8 (i.e., 60% of EV=17.2 m.min^-1^), respectively. Therefore, the energy that sustains the exhaustive test effort is predominantly from anaerobic metabolism. Consequently, even with aerobic characteristics, Ferreira’s protocol [12] improved aerobic and anaerobic performance parameters.

Similar to Pereira et al.’ study [12], after 4 weeks of downhill running sessions at a grade of −14%, mice had diminished exhaustion time, exhaustion velocity, and time to exhaustion by approximately 43.7%, 30.2% and 55.0%, respectively. The major findings of the present investigation were DNA damage in peripheral blood and skeletal muscle cells in trained and overtrained mice, and their relationship with GSH and TBARS levels in skeletal muscle cells, and GSH levels in total blood. Interestingly, independent from the experimental group, we found significant correlations between the comet assay scores and GSH levels measured in blood and muscle cells. These data are in accordance with other investigations [16,17] showing that changes in muscle are reflected in the blood.

Our study found that Ferreira’s aerobic training protocol [12] was able to improve performance in mice without leading to DNA damage in peripheral blood and skeletal muscle cells. Furthermore, after 8 weeks of training, mice did not have significantly different levels of GSH and TBARS in skeletal muscle cells compared with the control group. However, trained mice had significantly lower levels of GSH in total blood compared with those in the control group. Using the same aerobic training protocol as in our study, Ferreira et al. [18] observed significantly higher levels of maximal citrate synthase activity in the gastrocnemius muscle, and a higher GSH/oxidized-glutathione (GSSG) ratio and total glutathione levels compared with untrained mice (i.e., GSH+2GSSG) in plantaris and soleus muscles.

Based on the present results in the trained group and in previous investigations [3,18], we conclude that our trained mice did not present with DNA damage in skeletal muscle cells because of a lack of significant changes in GSH and TBARS levels in the same tissue. However, we are not able to explain why trained mice did not have DNA damage in peripheral blood cells, even with lower levels of GSH in total blood. We speculate that lower GSH levels only are not sufficient to induce DNA damage in blood cells. However, overtrained mice had DNA damage in peripheral blood and skeletal muscle cells, as well as lower GSH levels in skeletal muscle cells and total blood, and higher TBARS levels in skeletal muscle cells compared with the control and trained groups.

The relationship between overtraining and oxidative stress has been previously studied in humans [19,20] and rodents [9,21,22]. Margonis et al. [19] observed that a 3-week overtraining period led to higher levels of TBARS (56%), catalase (96%), and GSSG (25%), and to lower levels of GSH (31%) and GSH/GSSG (56%) in 12 participants. Furthermore, another study on seven subjects who were severely overtrained concluded that increased oxidative stress plays an important role in the pathophysiology of overtraining syndrome [20]. Dong et al. [9] studied the protocol described by Hohl et al. [8] and considered that overtraining can activate nicotinamide-adenine dinucleotide phosphate oxidase-mediated overproduction of reactive oxygen species, inducing increased lipid peroxidation. Zoppi and Macedo [21] analyzed the levels of TBARS, reactive carbonylated derivates, GSH, catalase, citrate synthase, and stress protein HSP72 in soleus, extensor digital longus, and semitendinosus muscles of overreaching rats. The authors found higher levels of TBARS, reactive carbonylated derivates, and HSP72 only in soleus samples compared with rats submitted to 8-week endurance training, and concluded that the oxidative stress-induced overreaching protocol is fiber type dependent.

A previous study found that the red gastrocnemius muscle of Wistar rats in NFOR had significantly lower citrate synthase activity compared with FOR, but was similar to that of the controls [22]. In addition, mitochondrial complex IV activity measured in NFOR Wistar rats was lower compared with that in control and FOR rats. The authors considered that this impaired mitochondrial adaptation in the NFOR group was associated with increased antioxidant enzyme activity and increased lipid peroxidation (i.e., in muscle and plasma) compared with the FOR group and controls. Our data showing that NFOR mice had DNA damage in peripheral blood and skeletal muscle cells due to lower GSH levels and higher TBARS levels in skeletal muscle cells, and lower GSH levels in total blood, are consistent with previous investigations [9,21,22].

Recently, Pereira et al. [23] showed that the current overtraining protocol based on downhill running sessions is linked to high concentrations of cytokines in serum, skeletal muscles, and the liver. Therefore, they concluded that the performance decrease induced by NFOR is associated with muscle damage and inflammation [23]. Because muscle damage and inflammation are related to blood and skeletal muscle oxidative stress [24], we speculate that our findings in the overtrained group of DNA damage in peripheral blood muscle cells, and oxidative stress in skeletal muscle cells and total blood, may simply be responses to muscle damage and inflammation [24].

## Conclusion

In summary, aerobic and anaerobic performance can be improved with training at MLSS during 8 weeks, without leading to DNA damage in peripheral blood and skeletal muscle cells or to oxidative stress in skeletal muscle cells. However, NFOR induced by downhill running training sessions is associated with DNA damage in peripheral blood and skeletal muscle cells, and to oxidative stress in skeletal muscle cells and total blood.

## Methods

### Experimental animals and metabolic parameters

Male Swiss mice from the Central Animal Facility of the Ribeirão Preto Campus of the University of Sao Paulo (USP) were maintained in individual cages with a controlled temperature (22±2°C) on a 12:12-h light–dark inverted cycle (light: 6 PM to 6 AM; dark: 6 AM to 6 PM) with food (Purina chow) and water *ad libitum*. All experiments were approved by the Ethics Committee of the USP. Eight-week-old Swiss mice were divided into three groups: control (C, n=10, sedentary mice), trained (TR, n=10, performed an aerobic training protocol), and overtrained (OTR, n=10, performed an overtraining protocol).

Body weight and food intake of all groups were recorded weekly. Food intake was determined by subtracting the final food weight (i.e., weight of food put in each individual cage after 1 week) from the initial food weight (i.e., weight of food put in each individual cage on Monday morning). C, TR, and OTR mice were manipulated and/or trained in a dark room between 6 to 8 AM [11].

### Incremental load test

First, mice were adapted to treadmill running (INSIGHT®, Ribeirão Preto, São Paulo, Brazil) for 5 days, for 10 min.day^-1^ at 3 m.min^-1^[11]. As described by Ferreira et al. [12], rodents performed the incremental load test with an initial intensity of 6 m.min^-1^ at 0% with increasing increments of 3 m.min^-1^ every 3 min until exhaustion, which was defined when mice touched the end of the treadmill five times in 1 min. The EV (m.min^-1^) of mice was used to prescribe the intensities of aerobic training and overtraining protocols.

### Aerobic training protocol

The 8-week aerobic training protocol was based on the study of Ferreira et al. [12] and each experimental week consisted of 5 days of training followed by 2 days of recovery. Table 1 summarizes the aerobic training protocol.

**Table 1:** Aerobic training protocol characteristics

**Week**	**Intensity (%EV)**	**Volume (min)**	**Daily sessions**	**Treadmill grade (%)**	**Recovery between sessions (h)**
1	60	15	1	0	24
2	60	30	1	0	24
3	60	45	1	0	24
4	60	60	1	0	24
5-8	60	60	1	0	24

### Overtraining protocol

The 8-week overtraining protocol was based on the study of Pereira et al. [11], and each experimental week of the OT protocol consisted of 5 days of training followed by 2 days of recovery. Table 2 summarizes the overtraining protocol.

**Table 2:** Overtraining protocol characteristics

**Week**	**Intensity (%EV)**	**Volume (min)**	**Daily sessions**	**Treadmill grade (%)**	**Recovery between sessions (h)**
1	60	15	1	0	24
2	60	30	1	0	24
3	60	45	1	0	24
4	60	60	1	0	24
5	60	60	1	−14	24
6	75	75	1	−14	24
7	90	90	1	−14	24
8	90	90	2	−14	4

### Performance evaluations

The incremental load test (i.e., exhaustion time and exhaustion velocity) and the exhaustive test (i.e., time to exhaustion) were used as performance evaluation parameters 24 and 48 h after the last training session. The incremental load test was performed at week 0, at the end of week 4, and at the end of week 8. Because of the high intensity and treadmill inclination, the exhaustive test was not performed at week 0.

### Exhaustive test

Twenty-four hours after the incremental load test, the rats ran at 36 m.min^-1^ with 8% treadmill grade until exhaustion [25-28], which was defined as when mice touched the end of the treadmill five times in 1 min. This value was recorded as the time to exhaustion.

### Muscle and total blood collection

Twenty-four hours after the exhaustive test, mice were anesthetized with an intraperitoneal injection of 2-2-2 tribromoethanol 2.5% (10–20 μL.g^-1^). As soon as anesthesia was ensured by the loss of pedal and corneal reflexes, gastrocnemius muscle of both hindlimbs was removed. The left gastrocnemius muscle was minced in chilled Hank’s solution and cell suspensions (80 μL) were used for the comet assay. Right gastrocnemius muscle was stored at −80°C for subsequent determination of GSH levels and lipid peroxidation, as measured by TBARS. Subsequently, total blood was collected from decapitation and used for the comet assay and determination of GSH levels.

### Comet assay of peripheral blood and skeletal muscle cells

The comet assay (pH > 13) was performed according to Singh et al. [29]. The viability of skeletal muscle cells was determined by the trypan blue dye (CAS 72-57-1; Sigma-Aldrich, St. Louis, MO, USA) exclusion method immediately before the comet assay. Briefly, 10 μL peripheral blood or 80 μL skeletal muscle cell suspensions were mixed with 180 μL or 240 μL, respectively, of a low melting point agarose (0.5%) (Invitrogen, Carlsbad, CA, USA). This was spread onto microscope slides precoated with normal melting point 1.5% agarose (Invitrogen) constituting a slide with two layers of agarose [30]. The cells were covered with a coverslip and maintained at 4°C for 10 min. Coverslips were removed from slides and immersed in a freshly prepared lysis solution consisting of 2.5 M NaCl, 100 mM ethylenediaminetetraacetic acid, 10% dimethylsulfoxide, 1% Triton X-100, and 10 mM Tris, pH 10, for 22 h at 4°C. After lysis, the slides were placed in an electrophoresis unit containing 300 mM NaOH and 1 mM EDTA at pH > 13 and left for 20 min to denature the DNA. Electrophoresis was run for 20 min at an electric field strength of 1 V.cm^-1^ (25 V and 300 mA). Slides were subsequently immersed in a neutralization buffer (0.4 M Tris–HCl, pH 7.5) for 5 min.

After being dried at ambient temperature, slides were fixed in ethanol for 2 min and stored until analysis. After removal from storage, each slide was stained with GelRed (1:10^4^) (Biotium, Hayward, CA, USA) and immediately analyzed. All of the steps were conducted in the dark or dimmed light. The comet observations were made at 400 × magnification using a fluorescence microscope (Zeiss, Axiostarplus®, Oberkochen, DE) equipped with an excitation filter of 510–560 nm and a 590-nm barrier. One hundred nucleoids per animal (50 nucleoids per slide) were analyzed using Comet Assay IV software (Perceptive Instruments, Haverhill, UK). While undamaged cells have an intact nucleus without a tail, damaged cells have the appearance of a comet. To measure DNA damage, the most common parameters analyzed are the percentage of DNA in the tail, tail moment, and tail length [31]. The percentage of DNA in the tail is generally defined as the fraction of DNA in the tail divided by the amount of DNA in the cell multiplied by 100, and this parameter appears to be the most linearly related to dose and the easiest to intuitively understand [31]. Therefore, the present results from the comet assay are expressed as the percentage of DNA in the tail.

### Determination of GSH levels in skeletal muscle cells and total blood

Briefly, gastrocnemius tissue (0.2 g) was homogenized in 2.0 mL ice-cold KCl 1.15%, and the homogenate was diluted in water (1:4), precipitated with 50% trichloroacetic acid (TCA), and centrifuged at 855 × g for 10 min. An aliquot (0.5 mL) of the supernatant was added to 2.0 mL Tris–EDTA buffer (0.2 M, pH 8.9) and 0.1 mL 5-5’-dithio-bis(2-nitrobenzoic acid) (DTNB) 0.01 M in methanol. The solution was incubated at room temperature for 15 min and read at 412 nm with a standard α-cysteine curve prepared using concentrations of 0.10, 0.04, 0.02, and 0.01 μmol.mL^-1^. The results are expressed as nmol GSH.mg^-1^ of protein. Protein values were determined by a modified Lowry method [32], which provides a linear photometric response. The samples were read at 650 nm.

GSH levels in total blood were measured using the method of Ellman [33] in which reduced-non protein thiols were analyzed. Whole blood (0.3 mL) was hemolyzed using 10% Triton X-100 (0.1 mL) and precipitated with 0.2 mL 20% TCA. After centrifugation at 2376 × g for 10 min, aliquots of the supernatant were treated with 10 mM DTNB (50 μL), and the reaction product was measured at 412 nm with a standard α-cysteine curve at concentrations of 0.01, 0.025, 0.05, 0.1, and 0.15 μM. GSH content was expressed as μmol/mL of blood.

### TBARS levels in skeletal muscle cells

TBARS levels were determined according to the method proposed by Buege and Aust [34]. Briefly, an aliquot (0.5 mL) of the same homogenate mentioned above was mixed with 1 mL thiobarbituric acid (TBA) + TCA reagent (3.7 g/L TBA + 15% TCA in 0.25 mol.L^-1^ HCl). The mixture was heated for 15 min in a boiling water bath. After cooling in an ice bath, the tubes were centrifuged at 855 × g for 15 min. The absorbance of the supernatant at 535 nm was measured, and the concentration of TBARS in the samples was determined using a standard curve made with freshly prepared 1,1,3,3-tetraethoxypropane at concentrations of 5.12, 10.25, 20.50, and 40.10 nmol.mL^-1^. TBARS concentrations in gastrocnemius are expressed as nmol TBARS.mg^-1^ of tissue.

### Statistical analysis

Results are expressed as mean ± standard error (SE). According to the Shapiro-Wilk W-test, the data were normally distributed and the homogeneity was confirmed by Levene’s test. Therefore, repeated measures analysis of variance was used to examine the effects of training and overtraining protocols on the studied parameters. The violations of sphericity were measured by the Maucheley’s sphericity test. When violated, data were corrected using Greenhouse-Geisser adjustment. When repeated measures analysis of variance indicated significance, Bonferroni’s post hoc test was performed. Correlations between the parameters measured in muscle and blood (i.e., comet assay scores and GSH levels) were determined using Pearson’s correlation coefficient. Statistical analyses were performed using Statistica 8.0 computer software (StatSoft®, Tulsa, OK, USA). All statistical analyses were two-sided and the significance level was set at *P* < 0.05.

## Competing interests

The authors declare that they have no competing interests.

## Authors’ contributions

BCP participated in the concept and design of the study, data acquisition, data analysis, and interpretation, and revised the final version of the manuscript. JRP, LMGA, and ECF participated in data acquisition, analysis, and interpretation, and revised the final version of the manuscript. MRA and VPV participated in data acquisition, analysis, and interpretation, and contributed to writing the Materials and Methods. ERR, CTS, and DEC significantly contributed to the Discussion and revised the final version of the manuscript. ASRS participated in the concept and design of the study, data acquisition, analysis, and interpretation, and wrote the manuscript. All authors read and approved the final manuscript.
